# Reverse signalling of membrane TNF in human natural killer cells: a comparison of the effect of certolizumab pegol and other anti-TNF agents

**DOI:** 10.1186/1479-5876-9-S2-O3

**Published:** 2011-11-23

**Authors:** Gianluca Fossati, Andrew M Nesbitt

**Affiliations:** 1 UCB Pharma, Slough, UK

## Introduction

Differences have been seen among the anti-TNFs in mediating reverse signalling of membrane TNF-α (mTNF-α). Natural killer (NK) cells express high levels of mTNF-α and may be involved in rheumatoid arthritis pathogenesis. We examined the effect of certolizumab pegol (CZP) and the other anti-TNFs, adalimumab (ADA), etanercept (ETA) and infliximab (IFX), on cellular activities of NK cells.

## Methods

Peripheral blood mononuclear cells from healthy volunteers were isolated and incubated in the presence of 100 U/mL recombinant human (rh) interleukin-2 (IL-2) for 20 h and 10 μg/mL anti-TNF agents or isotypic controls (human IgG_1k_ and Fab′ PEG) for a further 4 h. Antibody-dependent cellular cytotoxicity (ADCC) was measured by loss of cell membrane integrity, by binding of 7-amino-actinomycin D to DNA. For analysis of soluble cytokine production and β-hexosaminidase release as an index of cell degranulation, NK cells were incubated with rhIL-2 and anti-TNF agents. The concentration of the soluble cytokine interferon-γ (IFN-γ) was determined by enzyme-linked immunosorbent assay. β-hexosaminidase release was quantified upon enzymatic cleavage of 4-methylumbelliferyl N-acetyl-β-D-glucosaminide in citrate buffer (0.1 M, pH 4.5) by spectrophotometric analysis.

## Results

When NK cells were incubated with anti-TNF agents in the presence of rhIL-2, IFN-γ production was significantly increased from the control level of ~84 pg/mL to ~1.1 ng/mL. All 4 anti-TNF agents stimulated NK cell degranulation, as measured by β-hexosaminidase levels, to a level of ~27% degranulation compared with a control level of 3%. ADCC measured in NK cells was detectable only with ADA, ETA and IFX (44.3%, 46.4% and 47.9%, respectively) and not with CZP (1%).

## Conclusion

Anti-TNF agents may result in increased NK cell–mediated cytotoxicity by promoting the release of multiple cytotoxic effector molecules and inflammatory cytokines via reverse signalling through constitutively expressed mTNF-α. CZP can activate NK cells but, in contrast to conventional anti-TNFs, does not mediate ADCC due to its unique structure (lacking an Fc region).

